# Mesenchymal Stem/Stromal Cell Production Compliant with Good Manufacturing Practice: Comparison between Bone Marrow, the Gold Standard Adult Source, and Wharton’s Jelly, an Extraembryonic Source

**DOI:** 10.3390/jcm8122207

**Published:** 2019-12-14

**Authors:** Caroline Laroye, Mélanie Gauthier, Hélène Antonot, Véronique Decot, Loïc Reppel, Danièle Bensoussan

**Affiliations:** 1 CHRU de Nancy, Unité de Thérapie Cellulaire et banque de tissus, 54500 Vandoeuvre-lès-Nancy, France; 2 CNRS, UMR 7365, 54500 Vandoeuvre-lès-Nancy, France; 3 Faculté de Pharmacie, Université de Lorraine, 54000 Nancy, France

**Keywords:** mesenchymal stem/stromal cells, bone marrow, Wharton’s jelly, good manufacturing practice

## Abstract

Many clinical trials report mesenchymal stem/stromal cells (MSCs) efficacy in various indications. Therefore, standardization of MSC production becomes necessary. MSC properties are impacted by tissue origin, especially if they are from extraembryonic tissue or adult sources. For this reason, we evaluated the impact of MSC tissue origin on production. Methods: Three productions of MSC from Wharton’s Jelly (WJ) or from bone marrow (BM) were performed according to good manufacturing practice. The identity (phenotype, differentiation, and clonogenic capacities), safety (karyotype, telomerase activity, sterility, and donor qualification), and functionality (viability, mixed lymphocyte reaction) of each cell batch were analyzed. Results: Slight differences between MSC sources were observed for phenotype, telomerase activity, and clonogenic capacities. Conclusion: Both sources have made it possible to quickly and easily obtain clinical grade MSC. However, as availability of the source is thought to be essential, WJ seems more advantageous than BM.

## 1. Introduction

For a decade, the enthusiasm surrounding mesenchymal stem/stromal cells (MSC) has been steadily increasing. Their capacity for proliferation, self-renewal, differentiation, and their important immunomodulation properties make them very attractive for clinical use [1]. Indeed, currently more than 400 clinical trials use MSC in various indications ranging from myocardial infarction [2], sepsis [3,4,5], Graft versus Host disease [6,7], or diabetes [8,9].

These cells with fibroblastic morphology were defined in 2006 by the international society for cellular therapy (ISCT) as (1) adherent to the cell culture plastic; (2) able to differentiate into osteocytes, chondrocytes, and adipocytes; (3) with a phenotype that is positive for the CD73 CD90 CD105 mesenchymal markers and negative for the CD34 CD45 HLA-DR hematopoietic markers. This definition has become essential due to the dramatic increase in MSC applications [10]. However, their heterogeneity makes the ISCT definition too general. Indeed, MSC properties and phenotype seem to be mainly influenced by their environment and, especially, by their tissue source. Many studies report immunomodulatory properties, proliferation, or differentiation depending on the source [11]. For example, bone marrow MSC (BM-MSC) present a lower proliferation potential than Wharton’s Jelly MSC (WJ-MSC); those from extraembryonic tissues present a lower capacity of differentiation into adipocytes compared to those from adult sources [12] or MSC from menstrual blood with higher antibacterial properties than those from adult sources like BM-MSC [13]. Variations of MSC characteristics depending on the source have recently led ISCT to recommend specifying the tissue origin of MSC, for example, BM-MSC for bone marrow MSC or UC-MSC for umbilical cord MSC, to highlight tissue-specific properties [14]. Therefore, it is essential to determine the most appropriate source of MSC to get the best-expected effect depending on the therapeutic application considered. Regarded as the gold standard source, bone marrow (BM) presents drawbacks in clinical grade production. The invasive and painful procedure for BM collection, including a general anesthesia, is an important barrier to donation. Similarly, the amount of MSC contained in a bone marrow donation remains relatively low (frequency). In addition, clinical protocols tend to use high MSC doses, ranging from 1 × 10^6^ to 10 × 10^6^ MSC/kg, therefore, the production must be consistent. In this sense, Wharton’s Jelly (WJ) seems very attractive due to abundant tissue source, easy and painless collection, and high MSC expansion potential.

MSC, as advanced therapy medicinal products, require production and quality controls in agreement with good manufacturing practice (GMP). The results of these quality controls are limiting—any result out of specifications would lead to the non-qualification of the MSC batch and consequently, to a financial loss. Thus, based on our experience of BM-MSC and WJ-MSC clinical productions, we sought to trace the feasibility and the difficulties associated with these types of productions for their clinical use.

## 2. Experimental Section

### 2.1. Collection and Production of BM-MSC

Bone marrow collection was performed after information was given to and consent was gained from the donor. The donation was realized in the context of a hematopoietic stem cell donation under general anesthesia. A maximum of 20 mL/kg was collected from the iliac crests and was filtered. Once the sample was received at the cell therapy unit, BM cells were counted and 64 × 10^6^ total nucleated cells were seeded in 250 mL of αMEM media enriched with 10% of platelet lysate (Macopharma, Mouvaux, France) in a cellstack (area surface 1272 cm^2^) (Corning, Macopharma, Mouvaux, France).

After two days, the media was changed to remove non-adherent cells. MSC were trypsinized (TrypLE™ Select CTS™ (1X), Fischer Scientific, Illkirch-Graffenstaden, France) at the end of passage 0 (P0) when a confluence of 80% was reached. Cells were plated using a closed system kit (Macopharma, France) at a density of 3000 MSC/cm^2^. The culture was continued up to P1 in normoxia (21% O_2_). Stopping the culture at passage 1 is explained by the early senescence of the MSC from the BM.

### 2.2. Collection and Production of WJ-MSC

Umbilical cords were collected at the Maternity Hospital of Nancy after pregnant mothers had signed an informed consent form in compliance with the French national legislation regarding human sample collection, manipulation, and personal data protection. This collection was approved by the Nancy Hospital ethics committee and French ministry of research (No. DC-2014-2114). All the WJ-MSC productions were performed in GMP conditions with α-MEM culture medium (Macopharma, France) enriched with 5% of Platelet lysate (Macopharma, France). Briefly, the umbilical cords were immersed for 1 h at room temperature in an antibiotic–antifungal solution composed of αMEM, Amphotericin B (0.05 g/L); Vancomycin (1 g/L); and Amoxicillin (1 g/L).

The cord was then cut into thin sections of 5 cm and the amniotic membrane was broken by gently passing the scalpel along the cord. Cross sections of the cord (2 to 3 mm) were then made. Each fragment was transferred one by one to a small flask (TPP, Trasadingen, Switzerland) and was allowed to attach to the plastic surface for 15 min before the addition of 30 mL of αMEM medium supplemented with 5% platelet lysate. The small flasks of cultures were incubated at 37°C in hypoxia (5% CO_2_, 5% O_2_), allowing for improvement of WJ-MSC capacities [12]. The medium was changed after 4 to 5 days of culture. After nearly 10 days of migration and cell adhesion, the cross sections of the cords were removed and the medium was renewed. When the confluence of the cells was up to 80%, the medium was removed and kept for the centrifugation step, small flasks were washed with PBS (Macopharma, France), trypsinization was performed for 5 min and the addition of the previously used medium was stopped, and cells were centrifuged and plated using closed system kits (Macopharma, France) into cellstacks for P1 at a density of 1000 MSC/cm^2^. WJ-MSC were cultured up to P3 (final product). The same culture conditions were applied for passage 1, 2, and 3.

### 2.3. Freezing and Thawing

After trypsinization, MSC were washed by centrifugation and the concentration was adjusted to a minimum of 1.10^6^ MSC/mL. A cryopreservation solution, previously cooled to 4°C, composed of 80% albumin and 20% Dimethyl sulfoxide (DMSO), was added to the cell suspension in equal volume and under gentle mixing. Freezing was performed through a controlled rate freezer (Planer Kryo, Cryo Products, Hertogenbosch, The Netherlands). MSC were stored in vapor phase nitrogen.

The MSC were thawed in a water bath at 40°C for 5 min and then washed by centrifugation with a solution composed of albumin 4% (Vialebex), NaCl 50%, and ACD 10%.

### 2.4. Quality Controls

#### 2.4.1. Infectious Markers

Biological donor screening was performed in compliance with the regulatory infectious markers for any HSC donation, including a viral genomic diagnosis for HIV, HBV, and HCV. Screening must be negative for HIV, HBV, HCV, HTLV, and syphilis. For EBV, CMV, and Toxoplasmosis, only IgG antibodies can be positive.

#### 2.4.2. Microbiological Controls

Aerobic and anaerobic bottles of blood cultures were seeded after collection and each time the medium was changed at the trypsinization step and before freezing. These controls were carried out at the Microbiology laboratory of the University Hospital of Nancy.

#### 2.4.3. Phenotype and Cell Count

Once 80% confluence was reached, MSC were washed with PBS and then detached by trypsin action (Macopharma, France). A total nuclear cell (TNC) count was performed with a cell counter (POCH100i, Sysmex, PCOH100i, Sysmex, Roissy CDG, France) while MSC were counted on Malassez.

To evaluate the expression of surface markers, 2 × 10^6^ MSC were labeled with a positive-cocktail of antibodies labelling mesenchymal markers and containing the anti-CD90, CD73, CD105 mAbs, and with a negative-cocktail of antibodies containing the anti-CD34, CD45, CD11b, CD19, HLA-DR mAbs (Stemflow hMSC Analysis kit, Becton Dickinson, Bergen County, NJ, USA). Viable cells were identified using the 7AAD marker of dead cells. The absence of contamination by endothelial cells was controlled by the endothelial marker CD144 for WJ-MSC.

#### 2.4.4. Mesodermal Differentiations

Adipogenic and osteogenic differentiations were performed according to the manufacturer’s instructions (Differentiation Media BulletKits, Lonza, Basel, Switzerland). To induce osteogenesis, MSC were seeded at 3.1 × 10^3^ cells/cm^2^ in complete medium in a 12-well plate. After 24 h, the Osteogenesis Induction Medium (Lonza) was added to the adherent cells, the medium was replaced twice a week, and the differentiation was carried on until 21 days. MSC control consisted cultivating the cells with only basal medium. At day 21, calcium mineralization was assessed by coloration with Alizarin Red (Sigma, St. Louis, MO, USA). Cells were washed in PBS and fixed in 70% ethanol for 30 minutes at 4°C, followed by two wash steps in H_2_O. Cells were stained in 40 mM Alizarin Red pH 4.2 for 5 minutes at room temperature, rinsed in H_2_O, and then air dried. Red staining was observed by light microscopy. For adipogenic differentiation, MSC were seeded at 2.1 × 10^4^ cells/cm^2^ in complete medium in a 12-well plate. At 100% confluence, three cycles of induction/maintenance were performed. Each cycle consisted of feeding MSC with supplemented Adipogenesis Induction Medium (Lonza) and culturing for 3 days, followed by 1 to 3 days of culture in supplemented Adipogenic Maintenance Medium (Lonza). After three complete cycles, MSC were incubated with Adipogenic Maintenance Medium until 21 days and the medium was replaced twice a week. MSC control consisted of culturing the cells, only with Adipogenic Maintenance Medium. After 21 days, a fluorescent staining with AdipoRed™ (Lonza) was performed to detect lipid droplets. Cells were washed with PBS and incubated with 2 μL AdipoRed™ per mL PBS for 15 min. Fluorescence was observed by confocal microscopy.

For chondrogenic differentiation, 2.5 × 10^5^ MSC were transferred into 15 mL conical tubes, centrifuged at 150 g for 5 min, and grown as high-density pellets for 28 days in chondrogenic medium containing DMEM-high glucose (Gibco, Grand Island, NY, USA) supplemented with glutamine 2 mM, penicillin 100 U/mL, streptomycin 100 μg/mL, amphotericin B 2.5 μg/mL, 1% Insulin-Transferrin-Selenium (BD Biosciences, San Jose, CA, USA), 0.1 μM dexamethasone (Sigma), 50 μg/mL ascorbate-2-phosphate (Sigma), 100 μg/mL sodium pyruvate (Sigma), 40 μg/mL L-proline (Sigma), and 10 ng/mL transforming growth factor β1 (TGF- β1) (Gibco). The medium was changed twice a week. MSC control consisted of culturing the cells only with basal medium. After 28 days, matrix protein synthesis was evaluated by histochemical analysis. The pellets were fixed, embedded in paraffin blocks, and cut into thick sections. Proteoglycans were stained by Alcian Blue.

#### 2.4.5. CFU-F

For colony-forming-unit fibroblast (CFU-F) assays, MSC were seeded in two T25 at 10 and 20 cells/cm^2^, respectively. They were cultured in complete medium, as previously described, for 10 days. Then, they were washed with PBS, fixed with ethanol, stained with Giemsa solution (Sigma), and rinsed with water. CFU-Fs of more than 50 cells were scored and data were expressed as the total colony number per 100 cells.

#### 2.4.6. Karyotype and Telomerase Activity

Karyotypes of MSC were realized after blocking the cell nuclei in metaphase. Twenty mitoses were analyzed. Telomerase activity was performed by qRT-PCR by the TRAP method (Telomere Repeat Amplification Protocol) in the cytogenetics laboratory of Clermont-Ferrand hospital. The results of real-time RT-PCR were expressed as normalized hTERT expression, i.e., the ratio between hTERT and ABL transcript numbers multiplied by 100. The samples with the absence of hTERT amplification and copy number of transcripts ABL >10,000 were considered negative for hTERT expression. All experiments were performed in triplicate, with good consistency of results.

#### 2.4.7. Mixed Lymphocyte Reaction (MLR)

The lack of immunostimulatory capacity of MSC has been monitored. For this purpose, lymphocyte proliferation tests were carried out with the kit: DELFIA^®^ Cell Proliferation kit (Perkin Elmer, Villebon-sur-Yvette, France). This immunofluorescence technique is based on the incorporation of a pyrimidine analogue, 5-bromo-2’-deoxyuridine (BrdU), into the newly synthesized DNA strands during cell proliferation. BrdU is detected using a monoclonal antibody labeled with europium. After removal of the unbound antibodies, the addition of a chelating solution allows the dissociation of the europium ions and the formation of a highly luminescent chelate of which its fluorescence will be proportional to the DNA synthesis. Further, 1 × 10^5^ PBMC from an allogeneic donor 1 (D1) were cultured for 3 days alone or in co-culture either with:

- 1 × 10^5^ allogeneic irradiated (25 Gy) PBMC from a second donor (D2 *), used as stimulating cells for positive control;
- 1 × 10^5^ MSC * irradiated (25 Gy) to test MSC immunogenicity;
- or 1 × 10^5^ PBMC D2 * irradiated (25 Gy) and 1 × 10^5^ MSC to test MSC immunomodulation capacities.

#### 2.4.8. Statistics

Data are presented as means ± SEM. Between group differences were tested by two-way ANOVA with Sidak correction or Student’s *t* test when appropriate. Analyses were performed using GraphPad Prism software.

## 3. Results

### 3.1. Production of MSC

**Figure 1:**
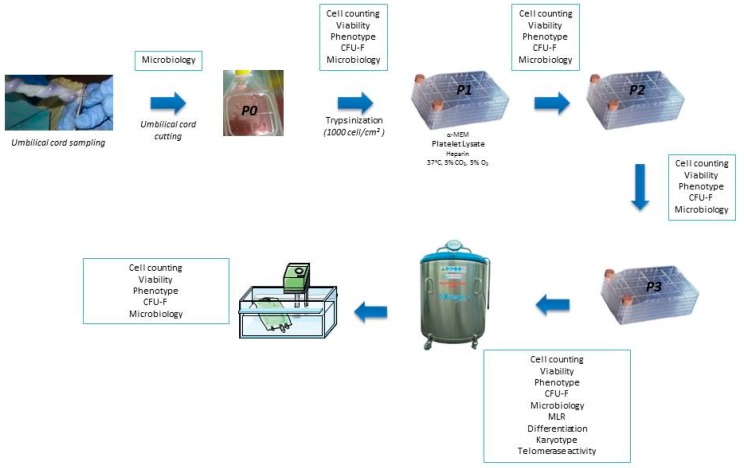
Wharton’s Jelly mesenchymal stem/stromal cells (MSC) production and quality controls.

Three MSC batches were produced from WJ of umbilical cord donors in a cord blood donation context. The MSC were cultured in a medium composed of αMEM (Macopharma) enriched with platelet lysate 5%. At each passage, different quality controls were carried out (Figure 1). The mean P0 duration was 24 +/− 2.2 days and the mean number of MSC obtained by small flask was 2 × 10^6^ +/− 0.9 cells. Seeding, carried out in a closed system using kits, was performed at the density of 1000 MSC/cm^2^ at the end of the P0 in 1 cellstack. The number of cells obtained by cellstack at the end of P3 was between 21.7 × 10^6^ and 29.55 × 10^6^, depending on the batch. The mean cumulative population doubling using the formula log N/log 2, where N is the cell number of the confluent monolayer divided by the initial number of cells seeded [15], during production was 4.58 +/− 0.19. The mean yield after thawing (cell number after thawing/cell number before freezing) was 61.3 +/− 4.5%.

**Figure 2:**
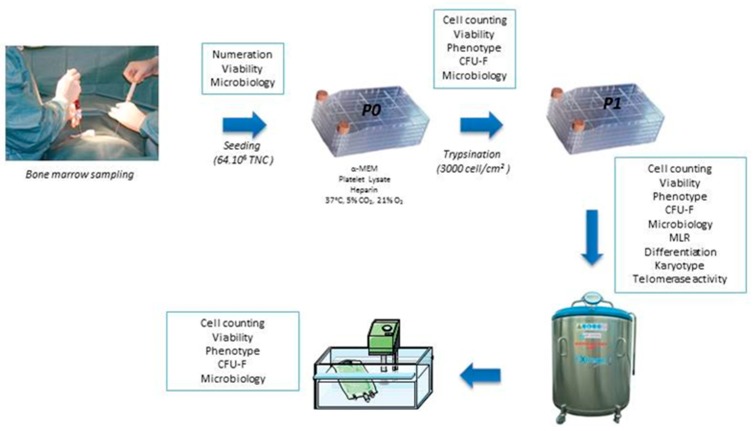
Bone marrow MSC production and quality controls.

Three batches were produced from bone marrow derived from HSC intrafamilial allograft donation. Donors gave their consent for the use of a sample of the collection (5–10 mL) to produce MSC (Figure 2). The TNC were cultured in a medium composed of αMEM enriched with 10% platelet lysate. The mean duration of P0 was 24 +/− 5.9 days and the mean number of MSC obtained by Cellstack was 37 × 10^6^ +/− 4.07 cells.

The cells were seeded at the end of the P0 in 3 to 4 cellstacks. The number of cells obtained by cellstack at the end of P1 was between 13.5 and 35.5 × 10^6^ according to the productions. The mean cumulative population doubling was 2.38 +/− 0.41. The mean yield after thawing was 75.3 +/− 13.4%.

### 3.2. Identity of MSC: Phenotype, Clonogenic, and Differentiation Capacities

**Figure 3:**
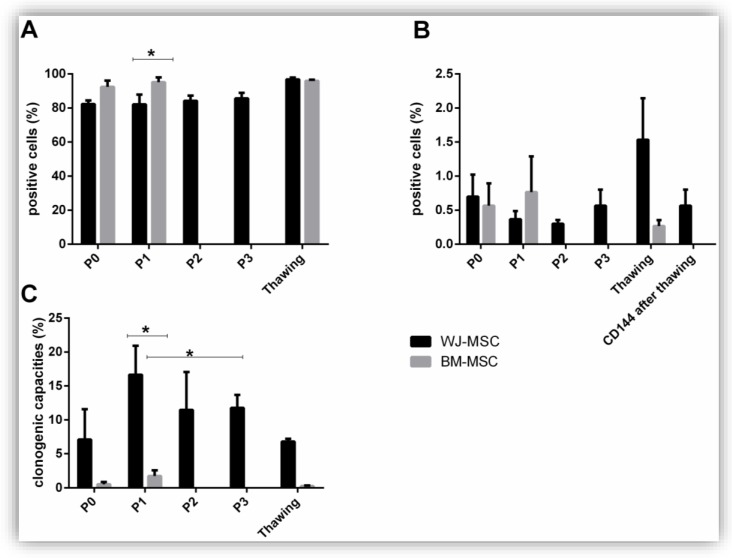
MSC characterization. Phenotype was analyzed using positive antibodies cocktail (anti-CD90; CD105; CD73) (**A**) and negative antibodies cocktail (anti-CD45, HLA-DR, CD34, CD19) (**B**). Clonogenic capacities were evaluated by colony-forming-unit fibroblast (CFU-F) culture (**C**). Results are expressed as mean ± SEM (*n* = 3 per group). * *p* <0.05.

Phenotype was analyzed by flow cytometry at the end of every passage. A positive cocktail of antibodies labelling mesenchymal markers was used (Figure 3A). On average, 83.6 +/− 1.67% of WJ-MSC and 93.7 +/− 2.2% BM-MSC expressed CD90, CD105, and CD73 mesenchymal markers regardless of the passage. A significant difference was observed between the mesenchymal markers expression between WJ-MSC P1 and BM-MSC P1 (82.2 +/− 5.77% versus 95.1 +/− 2.91%). However, if we consider the final product before freezing, WJ-MSC P3 and BM-MSC P1, no significant difference was observed.

After thawing, mesenchymal markers were found at 96 +/− 1.08% and 96.7 +/− 0.5% on WJ-MSC and BM-MSC, respectively. No significant difference was found between mesenchymal markers before and after thawing.

Hematopoietic markers (CD45, HLA-DR, CD34, and CD19) were analyzed by a negative cocktail (Figure 3B). It was observed that, regardless of the passage, 0.48+/− 0.1% and 0.67+/− 0.28% of the cells expressed the hematopoietic markers when they were respectively isolated from the WJ and the BM. After thawing, 1.53 +/− 0.61% of WJ-MSC and 0.26 +/− 0.09% of BM-MSC were positive for hematopoietic markers. No significant difference was found.

One of the characteristics of MSC is their ability to form in vitro cell clones. This test determines the MSC capacity to divide and generate in vitro fibroblastic-type colony-forming units (CFU-F). Clonogenic capacities of WJ-MSC were 11.75 +/− 2.08%, while BM-MSC clonogenic capacities were 1.13 +/− 0.49% (Figure 3C). A significant difference in clonogenic capacities was observed between the WJ-MSC P1 versus BM-MSC P1 (16.6% versus 1.7%) and between the final products before freezing (WJ-MSC P3 11.77% versus BM-MSC P1 1.7%). After freezing, a decrease in clonogenic capacities was observed regardless of the source without any significant difference between WJ-MSC and BM-MSC.

**Figure 4:**
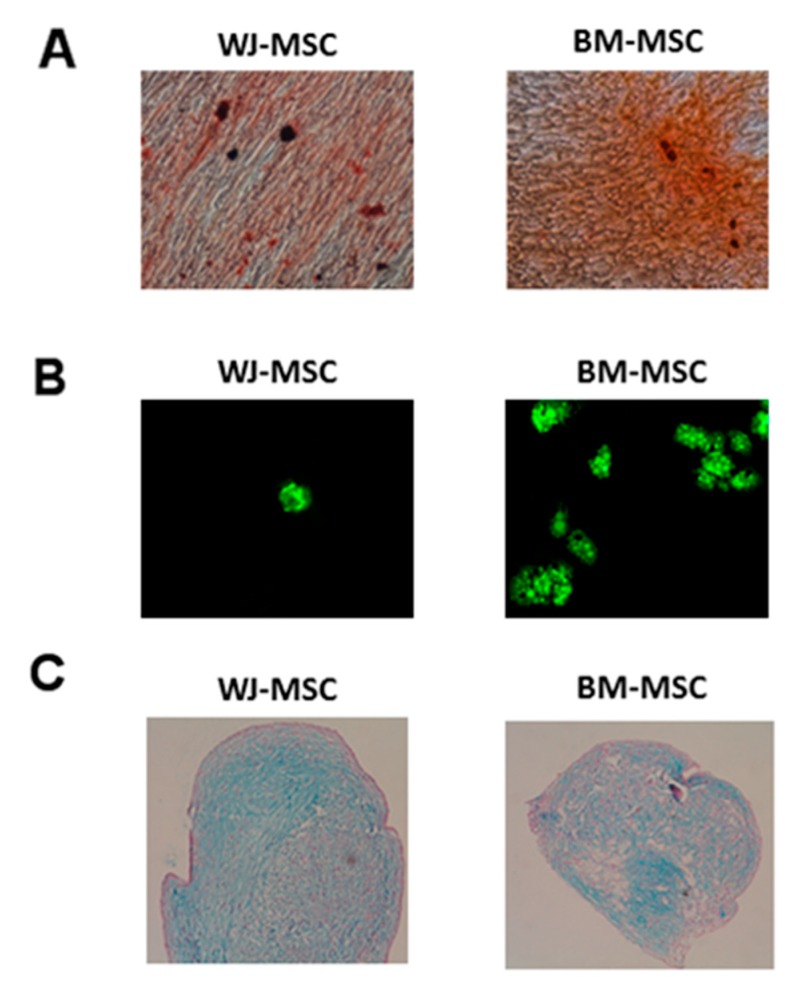
Differentiation of MSC depending on the tissue source after good manufacturing practice (GMP) production. (**A**) Osteogenic differentiation (Alizarin red; × 10 magnification); (**B**) Adipogenic differentiation (Adipored; × 10 magnification); (**C**) Chondrogenic differentiation (Alcian blue; × 10 magnification).

The osteogenic and adipogenic differentiations of WJ-MSC and BM-MSC were carried out for 21 days in contact with Lonza’s Differentiation media BulletKits medium. The revelations of calcium deposits and lipid vesicles were made, respectively, by red Alizarin (Sigma) and AdipoRed (Lonza). Osteogenic differentiation was obtained for all WJ-MSC and BM-MSC productions (Figure 4A). No difference was observed between the two sources. Adipogenic differentiation was obtained for all WJ-MSC and BM-MSC productions. However, adipogenic differentiation appeared weaker from WJ-MSC than that obtained by BM-MSC (Figure 4B). After 28 days, proteoglycan formations were observed both with BM-MSC and WJ-MSC without any difference between the tissue source (Figure 4C).

### 3.3. Safety of MSC: Immunogenicity, Microbiology, Serology, Karyotype, and Telomerase Activity

**Figure 5:**
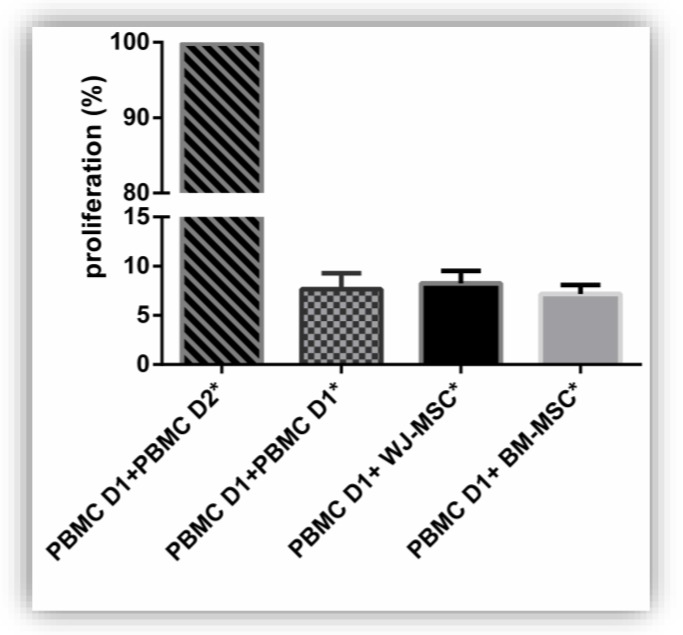
MSC source has no impact on immunogenicity. Wharton’s Jelly MSC (WJ-MSC) and bone marrow MSC (BM-MSC) immunogenicity was evaluated by a mixed lymphocyte reaction before thawing. (*) symbol means cells were irradiated (25 Gy). No significant difference was observed between the groups. Results are expressed as mean ± SEM (*n* = 3 per group).

In order to ensure the safety of the productions, various controls were carried out. Among these, a mixed lymphocyte reaction (MLR) test was performed to ensure the lack of MSC immunogenicity (Figure 5). PBMC proliferation from a healthy donor was 8.26% in co-culture with WJ-MSC and 7.12% in the presence of BM-MSC. No significant difference was found between the groups PBMC D1 + WJ-MSC, PBMC D1 + BM-MSC and the negative control PBMC D1 + PBMC D1 *.

Infectious markers from the donors were negative. No contamination of the product was found regardless of the source of MSC or the production stage. Similarly, the analysis of the final product karyotypes before freezing, WJ-MSC P3 and BM-MSC P1, highlights the absence of aneuploidy. Telomerase activity was never detected except for one WJ-MSC production at a very low level: 0.31 (Table 1).

### 3.4. Functionality of MSC: Viability and Immunomodulation Capacities

**Figure 6:**
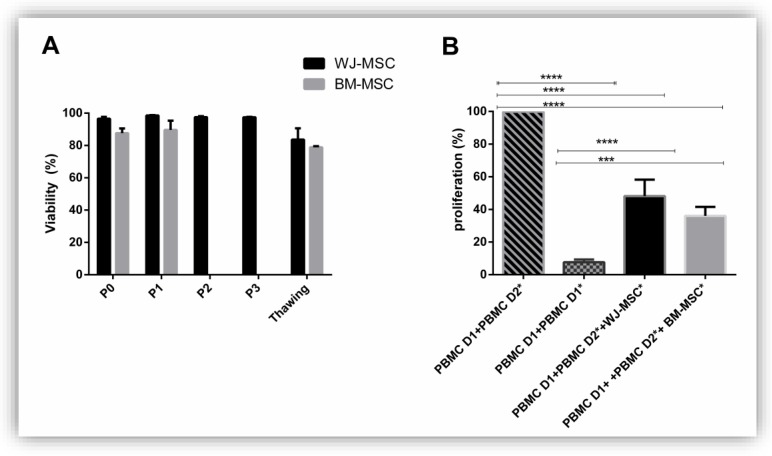
MSC source has no impact on viability and immunomodulation. WJ-MSC and BM-MSC viability was determined at each passage before and after freezing (**A**); WJ-MSC and BM-MSC immunomodulation was evaluated by a mixed lymphocyte reaction before freezing (**B**). Results are expressed as mean ± SEM (*n* = 3 per group). * *p* <0.05; *** *p* <0.001; **** *p* <0.001.

To analyze the functionality of MSC, we first determined their viability. We observed, regardless of the passage considered, a decrease in BM-MSC viability compared to the WJ-MSC viability. However, this difference was not significant (Figure 6A).

Evaluation of the immunomodulatory properties of MSC is interesting for measuring MSC efficiency (Figure 6B). Therefore, an MLR was realized. A PBMC D1 co-culture with PBMC D2 *, unrelated to donor 1 and previously irradiated in order to inhibit their proliferation, and MSC derived from BM or WJ was done. We observed a significant decrease in PBMC D1 proliferation when they were co-cultured in the presence of MSC regardless of their original tissue (PBMC D1 + PBMC D2 * 100% proliferation; PBMC D1 + PBMC D2 * + WJ-MSC 48.16 +/− 10.04% proliferation; PBMC D1 + PBMC D2 * + BM-MSC 36 +/− 5.5% proliferation). No significant differences were observed between PBMC D1 + PBMC D2 + BM-MSC and PBMC D1 + PBMC D2 * + WJ-MSC.

## 4. Discussion

Research and clinical trials around MSC are continuously increasing, resulting in the need for standardization of production methods. In a previous study, we observed slightly different effects of MSC depending on their tissue source in a murine model of septic shock [16]. We have concluded that WJ-MSC were at least as effective in the indication of sepsis and septic shock as BM-MSC. Here, we focused on the MSC production difficulties depending on their tissue source. Thus, based on our experience of clinical productions, we report the differences existing between MSC production derived from WJ and those from the BM.

However, this comparative study is limited by different culture conditions: WJ-MSC were cultivated in hypoxia up to P3 while BM-MSC were cultivated in normoxia until P1. These differences can be explained by earlier senescence of adult MSC [17]. Indeed, the telomere length is directly correlated to the age of the donor and to the time of culture before the senescence [18]. Therefore, the use of MSC from Wharton’s jelly allows a longer culture than when MSCs come from the BM.

Regarding normoxia, it was the gold standard of culture when the first production was initiated. Subsequently, normoxia was cancelled in favor of hypoxia. MSC are found in niches with low oxygen concentration keeping them in an undifferentiated state. MSC culture in hypoxic conditions can mimic these physiological conditions and improve their capacities compared to normoxic conditions [19]. Indeed, it has been shown that culture in hypoxic conditions allows a better proliferation, cell survival, secretion, and immunomodulatory activities [20]. Conversely, some studies have shown a deleterious impact of hypoxia on the properties of MSC. For example, the antibacterial abilities of MSC appear to be adversely affected by hypoxia [21]. Consequently, if hypoxic conditions seem to be an advantage for MSC production by reducing time production, it will be essential to adapt the culture conditions to future clinical use.

This study highlights some differences in terms of quality controls. In particular, we found that WJ-MSC expressed less mesenchymal markers than BM-MSC. However, this difference was significant only between WJ-MSC P1 and BM-MSC P1. In the final products (BM-MSC P1 and WJ-MSC P3), no significant difference was noted. According to the MSC specifications described by Lechanteur et al. and to the information on MSC specification in the approved European Blood and Marrow Transplantation center producing MSC, mesenchymal markers must be greater than 70% [22,23]. However, the question of cell purity can arise. If 70% of the cells express mesenchymal markers, 30% do not. However, both cells are infused. In an autologous context, this specification is acceptable, however in an allogeneic context, we recommend strictly following ISCT criteria (>95% expression mesenchymal markers; <2% for hematopoietic markers) for the final product before infusion into the patient. Here, contamination by endothelial cells may be considered due to the non-withdrawal of the umbilical vessels. Thus, to improve our practices, we added an endothelial marker, the CD144, which was not included in the definition of the ISCT. In this study, all the WJ-MSC productions were CD144 negative, attesting for an absence of contamination by endothelial cells. However, we recommend a CD144 expression specification less than 5% for WJ-MSC, like hematopoietic markers.

This study also revealed a significant difference in the clonogenic capacities of WJ-MSC and BM-MSC. This observation highlights the more primitive behavior of WJ-MSC than BM-MSC. This finding was confirmed by the analysis of hTERT activity. On three productions of WJ-MSC, one showed a telomerase activity. On the other hand, no telomerase activity was found in any BM-MSC production. A low telomerase activity can be found in extraembryonic MSCs and depending on the source [24]. Trivanovic et al. have demonstrated that mRNA hTERT levels vary depending on MSC source [25]. Telomerase activity is stable and no increase during culture suggests the safety of the culture [24]. Moreover, most studies describe a negative telomerase activity, which implies that the frequency of productions with a telomerase activity is low [26,27,28].

However, high hTERT activity can be due to unregulated growth or even tumor formation. Therefore, it may be difficult to argue to regulatory agencies the use of MSC with telomerase activity in clinical trials. Although rare, a production with low telomerase activity can be observed, as we experienced. Therefore, we recommend that WJ-MSC production centers analyze the telomerase activity at an early stage: hTERT analysis performed at the end of P0 and before freezing will avoid a long and expensive culture.

Concerning the characterization of our productions, adipogenic and osteocyte differentiation was obtained for all WJ-MSC and BM-MSC productions. However, adipogenic differentiation was less important with WJ-MSC. This feature has already been described and is probably related to the impregnation of 17β estradiol. In fact, 17β estradiol interferes with PPARγ, a transcription factor involved in adipogenic differentiation, which leads to a decrease in the expression of adipocyte-specific genes and inhibits adipogenesis [12,29]. This differentiation is necessary to respond to ISCT definition but is, however, unlike osteogenic or chondrogenic differentiations, not used in the current clinical MSC indications.

Finally, the amount of MSC obtained from WJ or BM was similar. However, the accessibility to MSC source must be considered. The number of BM donors is limited. Indeed, general anesthesia is required and the risk of infection and pain may discourage donors. Moreover, the regulatory status of MSC must not be lost sight of. MSC are defined as advanced therapy medicinal products [30]. Therefore, marketing authorizations can be obtained and MSC will be interesting for the pharmaceutical industry. Once the effectiveness of MSC has been demonstrated by academic research, patents will be bought by larger industries as was the case for CAR-Tcells [31]. Accessibility to the MSC source will then become a major issue for commercialization. Currently, BM-MSC production is carried out in the context of a HSCT family donation. However, it seems unlikely that a donor would agree to give his BM donation that is intended for a member of his family to a big pharma. The use of unrelated grafts also seems compromised. The last resort could be voluntary donors giving BM samples directly to the big pharma in exchange for compensation. Thus, bone marrow can quickly become an obsolete source due to the low number of potential donors, which is incompatible with large-scale production.

## 5. Conclusions

The clinical grade production of MSC from bone marrow and Wharton jelly is simple, relatively fast, and compliant with specifications. However, the accessibility of umbilical cord donations leads us to recommend this tissue source for GMP production.

## Figures and Tables

**Table 1:** Safety controls of MSC depending on the source.

	WJ-MSC			BM-MSC		
Infectious markers	Negative	Negative	Negative	Negative	Negative	Negative
Microbiology	Negative	Negative	Negative	Negative	Negative	Negative
Karyotype (46 XX or XY)	Normal	Normal	Normal	Normal	Normal	Normal
Telomerase activity %	0%	0.31%	0%	0%	0%	0%
